# Genotyping blastoderms of avian eggs

**DOI:** 10.1002/ece3.10821

**Published:** 2023-12-13

**Authors:** Kim Teltscher, Bart Kempenaers

**Affiliations:** ^1^ Department of Ornithology Max Planck Institute for Biological Intelligence Seewiesen Germany

**Keywords:** birds, genotyping, infertile eggs, sexing, sperm, undeveloped eggs

## Abstract

Undeveloped eggs occur frequently in birds and are often considered infertile, and discarded. However, the majority of undeveloped eggs may in fact have been fertilised and embryos might have died at an early stage. Such eggs contain valuable information, for example about offspring sex and paternity, and level of inbreeding. Obtaining such information may also give insight into the patterns and causes of early embryo mortality. Here we describe a simple technique for removing embryo cells from the blastoderm to obtain DNA to genotype the offspring and unequivocally ascertain fertilisation status, while retaining the overlying perivitelline layer (PVL) for sperm counts over the entire membrane. We tested this method on freshly collected eggs (high‐quality material), as well as on eggs from abandoned clutches and unhatched eggs (potentially deteriorated material) of blue tits (*Cyanistes caeruleus*). We sampled a total of 707 eggs from a wild population of blue tits, extracted DNA from the eggs' blastoderm using a Qiagen kit, and genotyped the samples with 14 polymorphic microsatellite markers, plus one sexing marker. Overall, we successfully genotyped 97% of all eggs. Our study is the most extensive dataset of genotyped undeveloped eggs to date and demonstrates that one can reliably genotype undeveloped fertile eggs as well as retain the PVL for observations of sperm and embryo cells.

## Graphical abstract

Undeveloped eggs occur frequently in birds and are often considered infertile, however, the majority of undeveloped eggs have in fact been fertilised and embryos have died at an early stage. Here we describe a simple technique for removing embryo cells from the blastoderm to obtain DNA to genotype and sex the offspring and retain the perivitelline membrane for observations of sperm and embryo cells.

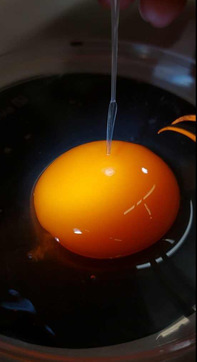

## 1 INTRODUCTION

In birds, eggs typically hatch after a period of incubation by one or both parents. However, for various reasons, hatching may fail. For example, eggs may fail to develop as a result of male or female infertility (Santema et al., 2020), or the embryo may die at an early stage due to genetic defects (Briskie & Mackintosh, 2004; Cordero et al., 2004; Heber & Briskie, 2010) or because of chemical pollution (Assersohn et al., 2021; Janssens et al., 2003). Eggs may also fail to hatch due to external causes, such as clutch abandonment due to the loss of a parent or after disturbance at the nest because they are physically damaged or as a result of unfavourable environmental conditions (Assersohn et al., 2021). Researchers can use these undeveloped eggs as a source of valuable data.

A recent study on great tits, *Parus major* and blue tits pointed out that an overwelming majority (99.9%) of unhatched eggs that showed no obvious signs of development and were therefore classified as infertile, were in fact fertilised whereby embryos died at an early stage of development (Hemmings & Evans, 2020). So far, it seems that few researchers have attempted to obtain genetic data from undeveloped eggs, and those who have tried, reported poor results. For example, Arnold and Griffiths (2003) describe problems with molecular sex identification of undeveloped eggs in a study on manipulation of egg sex ratios in jackdaws *Corvus monedula*, stating ‘…one limitation of this study, as with many studies of this nature, is that we did not know the sex of unhatched eggs’. In another example, Cichon et al. (2005) who investigated sex ratios of unhatched eggs in great tits, blue tits and collared flycatchers *Ficedula hypoleuca*, report that from 270 unhatched eggs, 45 (16%) showed no signs of development and that ‘The eggs showing no signs of development were considered unfertilised and no attempts were made to extract DNA from such eggs to avoid analysing DNA from blastoderms’. A third example is a study on herring gulls *Larus argentatus* where Egloff et al. (2009) demonstrated that 0–3 day old blastoderms from artificially incubated, nonviable eggs with no obvious signs of development could provide some DNA for genotyping. However, their results are minimal, based on a sample size of only nine eggs genotyped with three microsatellite markers, of which in most cases only one (seven eggs), and maximum of two (two eggs) loci were successfully amplified. Amplification of material from naturally incubated, non‐viable eggs proved to be even less successful, as they write ‘…amplification at any microsatellite locus was seldom achievable for egg contents that had no obvious signs of fertilization, due to difficulties in dissecting and extracting DNA from the blastodisc’.

A few studies have described methods to examine eggs that failed to hatch after the normal incubation period (unhatched eggs) with the aim to distinguish between infertile eggs and those in which embryo mortality occurred at an early stage of development (Birkhead et al., 2008; Hemmings & Evans, 2020). These studies suggest to examine the germinal disk (GD), and the perivitelline layers for embryo cells, sperm cells and holes through which sperm has passed (after the acrosome reaction; note that in birds multiple sperm can pass through the perivitelline membranes), but they do not describe genetic analyses, which could provide considerably more information. A recent study on the endangered hihi, *Notiomystis cincta* (Morland et al., 2023) describes a method similar to ours for genotyping solely unhatched eggs.

Obtaining genetic data from unhatched eggs is particularly important in paternity studies because the conclusions may change if all offspring can be genotyped. For example, in a study investigating inbreeding avoidance in purple‐crowned fairy‐wrens, *Malurus coronatus*, Kingma et al. (2013) wrote ‘Because eggs from many broods were not genotyped (due to difficulty genotyping unhatched eggs), we could not correct for paternity’.

In birds, the ovum is fertilised in the infundibulum of the female and the egg is laid approximately 24 h later. This means that by the time of laying many cell divisions have occurred to form the blastoderm which contains tens of thousands of cells (Birkhead et al., 2008). Thus, at laying, eggs should contain sufficient embryonic material for DNA extraction and PCR even if no further development should occur afterwards (see Aslam et al. (2012), who successfully sexed the blastoderms of unincubated eggs).

Here, we describe a quick and easy method to remove sufficient numbers of cells from the blastoderm for DNA extraction, while avoiding damage to the perivitelline layer (PVL) overlying it. Our method thus preserves the entire PVL and allows complete sperm counts, which is relevant for studies on copulation behaviour and fertilisation of the egg (e.g. Croyle et al., 2015; Santema et al., 2020, 2021) or on sperm morphology (Bennison et al., 2015; Hemmings et al., 2016). We applied this technique to 707 blue tit eggs from 258 nests and present the results by reporting various indicators of genotyping success and by comparing the genotypes of the embryo material to that of the known social parents.

Methods for removing the blastoderm from eggs already exist. For example, Gupta and Bakst (1993) describe a technique in which a doughnut‐shaped filter paper ring is placed on the yolk over the GD. The PVL is then cut around the filter paper and the ring with the PVL is carefully peeled off the yolk. Any adhering yolk is washed off and the blastoderm and PVL can be separated and prepared for microscopic examination (see also Aslam et al., 2012; Birkhead et al., 2008). The advantages of the doughnut filter paper technique are that the PVL of the GD is isolated and can be examined for sperm and holes and that the blastoderm can be removed relatively undisturbed such that the disposition of the cells can be retained, for example, for morphology studies. However, the main disadvantage of this technique is that it is time‐consuming such that examining large quantities of eggs is prohibitively laborious, in particular for small eggs.

Other methods described include sucking up the blastoderm with a pipette (Strausberger & Ashley, 2001), or simply cutting out the blastoderm and PVL (without filter paper; Egloff et al., 2009; Morland et al., 2023). These methods provide a faster, simpler technique for removing the blastoderm, but the main disadvantage is that the PVL over the GD—an area that tends to contain higher numbers of sperm relative to the rest of the PVL (Birkhead et al., 1994) does not remain intact. Another disadvantage is that including the PVL in the DNA sample can increase the risk of contamination of parental DNA, in particular when embryo DNA concentrations are very low (Arnold, Orr, & Griffiths, 2003; Morland et al., 2023).

## 2 MATERIALS AND METHODS

### 2.1 Study system

We conducted this study on a population of blue tits breeding in nest‐boxes in a natural mixed‐decidious forest (Reiherschlag, part of Westerholz forest, southern Germany; 48°08′26″ N 10°53′29″ E). The 35‐ha study site contains 277 nest‐boxes and the population has been monitored since 2007.

We caught all adults in the population in winter and in early spring, or while they were feeding 8–10 day old nestlings. We banded, measured and weighed each individual, took a 10–50 μL blood sample and implanted a passive‐integrated transponder (PIT‐Tag) under the skin on the back. We checked all nest‐boxes at least weekly prior to and during nest building and daily close to the start of laying and throughout the laying period. We identified the social parents of each nest by a combination of capture data and data from a PIT‐tag reader that was installed in each nest box (Schlicht et al., 2023).

The study was carried out in accordance with the German Animal Welfare Act (Deutsches Tierschutzgesetz). Permits were obtained from the Bavarian regional office for forestry LWF.

### 2.2 Egg collection

We collected two main types of eggs.

#### 2.2.1 Experimental eggs

In 2014 and 2019, as part of a project on egg content (Valcu et al., 2019), we collected a subset of eggs from experimental clutches. Each egg was marked with a number reflecting the order of laying. In 2014, we collected the first and ninth egg from 39 first clutches and from 10 of these clutches we also collected egg number two and every second egg laid thereafter, that is, eggs 1, 2, 3, 5, 7, 9 etc. continuing until the clutch was complete (*N* = 124 eggs). In 2019, we collected the first and fifth egg from all first clutches (*N* = 364). We collected each egg within a few hours after it was laid and replaced it with a dummy egg. In total, we collected 488 eggs, of which 481 were used in this study (124 from 2014 and 357 from 2019). Collected eggs were brought to the laboratory and all but 10 were kept in an incubator at 12°C until the next day and then incubated at 38°C, 60% humidity for exactly 24 h. We incubated the eggs to potentially increase the number of embryo cells, thereby increasing the DNA yield for improved genotyping results. Eggs were processed immediately after incubation, as described below. The remaining 10 eggs (second‐laid eggs from 2014) were kept in an incubator at 12°C for 2–3 weeks and then processed without further incubation at 38°C. Thus, we used these eggs (‘control’ eggs) to investigate how longer‐term storage without incubation (at 38°C) affected our success at obtaining sufficient DNA for genotyping and at counting sperm on the PVL.

#### 2.2.2 Non‐experimental eggs

We opportunistically collected three types of eggs: those that were abandoned by the parents (*N* = 206 eggs), those that did not hatch after the normal incubation period (*N* = 20) and those that were broken (*N* = 10). Abandoned clutches were collected after approximately 7 days of inactivity at the nest, determined as follows: First, we noted the day on which the last egg had been laid, then, we used the data from the PIT‐tag readers to determine whether the presumed social parents (those present at the nest earlier) still visited the nest. If not, we defined the nest as abandoned and collected the eggs. Clutch size of abandoned nests ranged from 1 to 12 eggs (*N* = 38 clutches). Thus, eggs varied in the time they had been in the nest before collection.

Eggs from abandoned nests were treated in different ways. Most eggs were brought to the lab and kept at room temperature for 1 to 7 days (*N* = 122 eggs) or for 11–19 days (*N* = 39 eggs), then incubated at 38°C for 24–48 h and finally stored in a fridge at 4°C for 0–24 h until processing. Another 45 eggs were stored at room temperature for 2–7 days and then processed without any incubation. Unhatched eggs (*N* = 20 from 6 clutches) were collected 5 days after the other eggs in the nest had hatched and kept at room temperature for 2–4 days before processing. Here, we only include unhatched eggs that showed no obvious signs of development. Eggs that were damaged during handling but still had intact yolks (‘broken eggs’, *N* = 10) were processed immediately on the day of collection.

### 2.3 Sample preparation

#### 2.3.1 Preparing the yolk

We opened each egg into a dry petri dish and isolated the yolk. If necessary, we rolled the yolk gently over with curved forceps until the germinal disc was lying on top. In 32 abandoned and two experimental eggs, the yolk broke. In these cases, we searched through the egg content to isolate the GD and salvage pieces of the PVL. In 80 eggs from the non‐experimental group (70 from abandoned clutches that were incubated at 38°C after collection, 10 unhatched eggs), we could not recover the PVL due to degradation, but we collected embryo tissue.

#### 2.3.2 Collecting embryo material

We placed the petri dish with the yolk under a binocular dissecting microscope at 6–10× magnification and illuminated it with a gooseneck lamp. Using micro‐scissors, we made an approximately 3 mm long incision in the PVL, parallel to the GD. Using a flat gel loading pipette tip (Fisher Scientific, 200 μL, 0.4 mm thickness) we gently scraped the blastoderm (see Burley & Vadehra, 1989) from underneath the GD while leaving the PVL intact. The pipette tip was then cut off and dropped into an Eppendorf tube with 100 μL 70% ethanol for subsequent DNA extraction. We repeated this with a new pipette tip until no more white material (area pellucida and area opaca) on the GD was visible (see Video 1).

**VIDEO 1:** So, I'm using a larger yolk here to demonstrate the procedure so it's easier to see. Here we have the germinal disc and I will take small scissors and cut the membrane just beside the germinal disc. And then I take a flat tip gel loading pipette tip and go under the membrane there and scrape off the blastoderm, which is the white material you can see on the germinal disc. I put the flat tip into ethanol and I take a new tip to remove any more of the blastoderm until all the white material is gone. So that looks good to me.

#### 2.3.3 Preparing the PVL

We cut the entire yolk in half with larger scissors, starting from the initial 3 mm cut, and picked up the two membrane halves with forceps. Each membrane piece was immediately washed in water to remove the yolk and then spread out on a microscope slide. We used water rather than PBS (phosphate‐buffered saline) because the latter often forms crystals on the PVL, obstructing visualisation of the sperm. We added a drop of Hoechst 33342 fluorescent dye and let the sample dry in the dark at room temperature. Slides were stored in a dark, dry place for up to 12 months before counting sperm.

#### 2.3.4 PVL inspection and sperm count

We examined the PVLs using a Zeiss Axio Imager.M2 fluorescent microscope with DAPI filter at 200× magnification and counted all sperm cells trapped on the PVL by systematically scanning the entire membrane (see Birkhead et al., 2008; Santema et al., 2021). After the removal of the blastoderm with the pipette tip, remaining embryo cells adhere to the PVL and may be dispersed during the washing process. Thus, we also recorded the presence of embryo nuclei, which are also stained bright fluorescent blue by the Hoechst 33342 on the PVL (Birkhead et al., 2008). Sperm counts were performed on 60% of all the eggs (255 experimental and 171 non‐experimental eggs).

### 2.4 Genotyping and parentage analysis

Embryo (blastoderm) material was prepared as described by Valcu et al. (2019). We extracted DNA from the samples using the QIAmp DNA Micro Kit from Qiagen (Cat. No. 56304) following the former protocol for Forensic Case Work samples (for details, see The QIAmp DNA Micro Handbook, Protocol: Isolation of Genomic DNA from Tissues). In brief, after removing the ethanol, we added 300 μL ATL buffer and 20 μL Proteinase K to the sample, mixed it by vortexing, and then incubated it at 56°C for 2 to 16 h. Then, we added 300 μL buffer AL, mixed the sample by vortexing, and incubated it at 70°C on a thermomixer with shaking for 10 min. We then centrifuged the sample at 16,000 *g* for 1 min and transferred it to a QIAmp MinElute column. This column was then centrifuged at 8500  *g* for 1 min. Afterwards, we discarded the flow‐through, placed the column in a new collection tube, pipetted 500 μL buffer AW1 onto the column and centrifuged it again at 8500  *g* for 1 min. We repeated the procedure (discarding the flow‐through and placing the column in a new collection tube), then added 500 μL buffer AW2, and centrifuged again at 8500  *g* for 1 min. Again, we discarded the flow‐through and placed the column in a new collection tube, which was then centrifuged at 16,000  *g* for 3 min to dry the column. We then discarded the collection tube, placed the column in a clean microcentrifuge tube, added 50 μL buffer AE to the column, incubated it at room temperature for 5 min, and finally centrifuged it at 16,000  *g* for 1 min. Samples that showed little or no amplification after the first PCR run (see below; *N* = 100) were concentrated using Amicon 100 K tubes, as follows: We added 300 μL PCR grade water to the DNA sample, pipetted the sample into the Amicon tube, centrifuged it at 14,000 *g* for 10 min, discarded the flow through and placed the sample tube turned upside down into a new microcentrifuge tube. We then centrifuged this tube at 1000 *g* for 2 min. The final volume of DNA sample recovered ranged from 16 to 30 μL.

We genotyped all samples at the sex‐linked marker P2/P8 (Griffiths et al., 1998) and at 14 polymorphic microsatellite markers: see tab. A1, p. 29 of Schlicht et al. (2023). For details of the PCR protocol, see Valcu et al. (2019). Each PCR reaction was run with a negative control, containing all components of the PCR except template DNA. No amplification was ever detected in the negative controls. We report the proportion of eggs that were successfully genotyped (we regarded 10 markers as the minimum number of loci to confidently assign parentage), the occurrence of genotyping errors (locus dropout and additional alleles) and the success in assigning parentage for nests with known social parents (using the software CERVUS; Kalinowski et al., 2007).

## 3 RESULTS

### 3.1 Sperm counts and fertilisation status

Macroscopic examination did not show a noticeable difference in the development of the blastoderms after 24 h of incubation compared to unincubated eggs.

Sperm and embryo cells were observed on all but 10 of the examined eggs, thus confirming that all these eggs were fertilised. One clutch of 10 eggs had neither embryo cells nor sperm on the PVL and no DNA could be amplified from the blastoderms. We discovered that these eggs came from a clutch of an infertile male (Santema et al., 2020). We excluded this clutch from the data set presented and discussed below.

The median number of sperm counted was 137 for experimental eggs (mean: 181, range: 8–1016) and 121 for non‐experimental eggs (mean: 160, range: 2–806; Figure 1).

**FIGURE 1:**
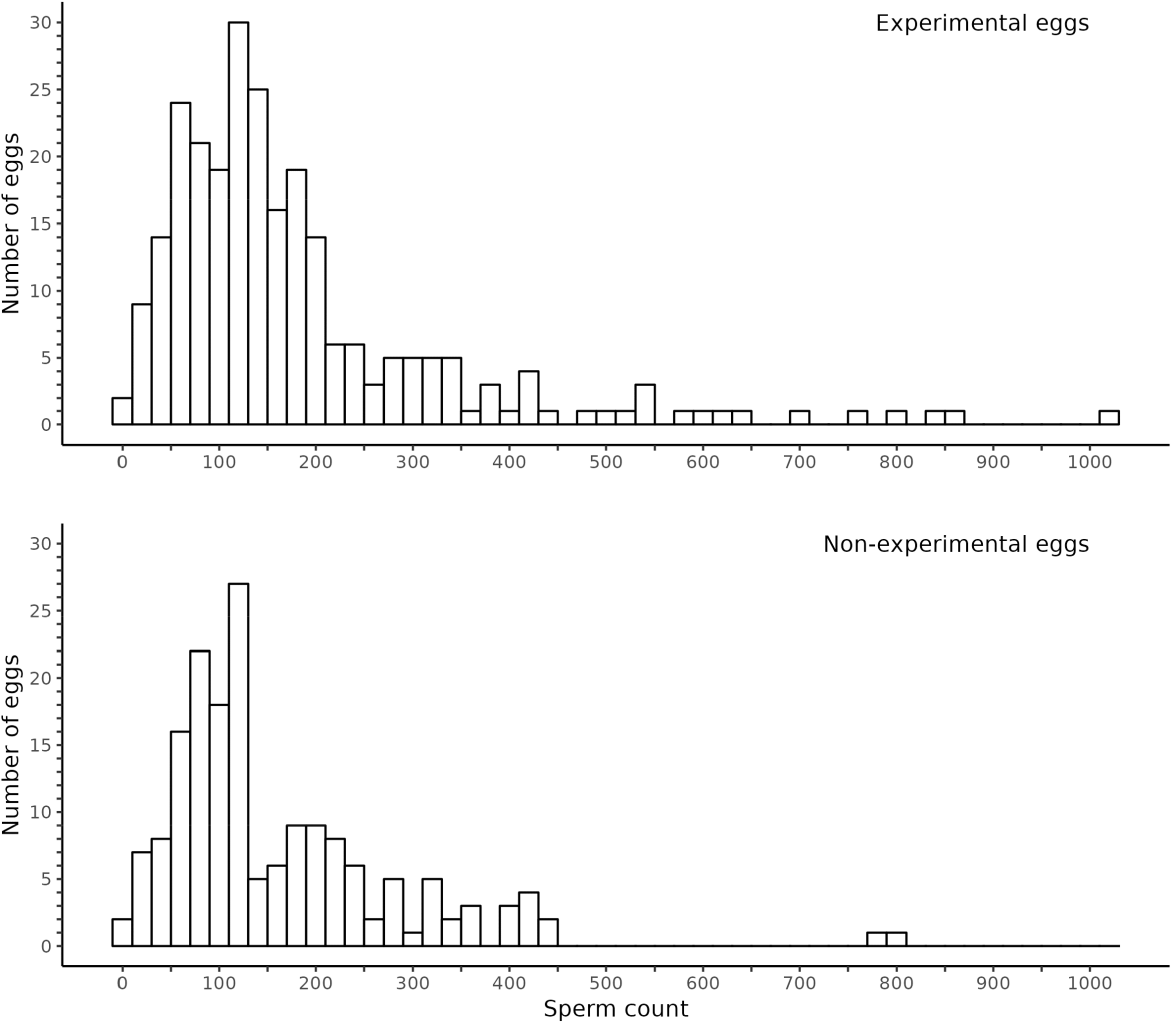
Number of sperm counted on the PVLs of experimental and non‐experimental eggs. To improve readability, we excluded one experimental egg that had an exceptionally high sperm count (2724).

### 3.2 Genotyping success rate

Most of the collected eggs could be successfully genotyped and sexed (Table 1). The sex ratio of the eggs (proportion of females: 0.53, *N* = 690) did not differ from the sex ratio of all blood‐sampled offspring collected during the same period (0.51, *N* = 1396 from 2014 and 2019; Fisher's Exact Test: *p* = .35). All 34 eggs where the yolk broke upon opening could be successfully genotyped, as well as all 10 experimental eggs (control eggs) from 2014 that had been stored at 12°C for 2–3 weeks without any incubation. However, one of these eggs amplified extra alleles (see below).

**TABLE 1:** Number and percentage of eggs that successfully amplified at a given number of loci for each egg category.

Egg type	*N*_total_	All 14 loci		>10 loci		0 loci		Sexing (P2P8)	
		*N*	%	*N*	%	*N*	%	*N*	%
Experimental	471	450	95.5%	469	99.5%	0		467	99.2%
Abandoned	206	174	84.5%	193	93.7%	3	1.5%	194	94.2%
Unhatched	10	5	50.0%	8	80.0%	0		9	90.0%
Broken	10	9	90.0%	10	100%	0		10	100%
Control	10	1	10.0%	9	90.0%	0		10	100%
Total	707	639	90.4%	689	97.4%	3	0.4%	690	97.6%

*Note*: Sexing (P2P8) locus shown separately.

### 3.3 Genotyping errors

Genotyping errors are not uncommon when working with low amounts of DNA (Taberlet et al., 1996; Taberlet & Luikart, 1999) We observed locus dropout (failure to amplify any alleles at a given locus) in 9% of all genotyped eggs (66/707), and 15 of these eggs (2%) were missing alleles at more than five loci (Table 2).

**TABLE 2:** The number of genotyped eggs that failed to amplify any alleles at a given number of loci (locus dropout) for different categories of eggs (see Section 2).

Egg type	Number of loci that failed to amplify alleles														
	1	2	3	4	5	6	7	8	9	10	11	12	13	14	15
Experimental	12	14	1	1		1									
Abandoned	4	10	1	4		3		1	2	2	2				3
Unhatched				2	1			1							
Broken		1													
Control															

We also occasionally detected more than the maximum of two expected alleles at a given locus. Overall, 10% (68/707) of the genotyped eggs had such extra alleles (Table 3). Morland et al. (2023) describe similar genotyping errors in their study on genotyping unhatched eggs of the hihi.

**TABLE 3:** The number of genotyped eggs for which extra alleles (3–4 per locus) were detected in a given number of loci for different categories of eggs (see Section 2).

Egg type	Number of eggs where >2 alleles were detected in						Total
	1 locus	2 loci	3 loci	4 loci	5 loci	6 loci	*N* (% of eggs)
Experimental	23	3	2	0	3	0	31 (7%)
Abandoned	16	6	2	1	2	0	27 (13%)
Unhatched	2	0	0	1	1	1	5 (50%)
Broken	2	0	0	0	0	0	2 (20%)
Control	1	1	0	0	1	0	3 (30%)
Total	44	10	4	2	7	1	68 (10%)

### 3.4 Parentage analysis

We selected a subset of eggs from nests for which both social parents were known (*N* = 606 eggs from 232 nests, 86% of all collected eggs). All but 10 eggs were genotyped successfully at 10 or more markers. In all cases, the social female was assigned as the genetic mother with high confidence, even in the 27 eggs with one (*N* = 20), two (*N* = 5) or three (*N* = 2) mismatches. The non‐exclusion probability for the first parent (mother) varied from 7.90 × 10^−4^ to 1.49 × 10^−8^ (mean ± SD: 5.39 × 10^−5^ ± 9.04 × 10^−5^). Paternity could be assigned with high confidence to 592 eggs: 474 eggs (80%) were sired by the female's social partner (the male at the nest) and 118 eggs (20%) were sired by an extra‐pair male. The non‐exclusion probability for the second parent (given a known genetic mother, *N* = 596 eggs) varied from 7.80 × 10^−4^ to 1.47 × 10^−14^ (mean ± SD: 1.92 × 10^−6^ ± 3.12 × 10^−5^). In all but four cases, the genetic father could be assigned with high certainty. The genetic father of four eggs could not be assigned, presumably because the extra‐pair male had not been sampled. Overall, we assigned parentage to 99.5% of the experimental eggs and 94.9% of the non‐experimental eggs.

## 4 DISCUSSION

It is generally assumed that eggs showing no obvious signs of development do not contain enough embryonic material to reliably sex or genotype. One study by Egloff et al. (2009) attempted to obtain embryo DNA from undeveloped eggs but with limited success. In many studies, unhatched, undeveloped eggs were simply discarded and no attempt was made to collect DNA (Arnold, Griffiths, et al., 2003; Cichon et al., 2005; Kingma et al., 2013). However, Aslam et al. (2012) describe a method to distinguish fertile from infertile eggs and show that fertile blastoderms contain enough DNA for reliable sexing of unincubated eggs.

We improve on this method by genotyping the fertile blastoderms of unincubated and slightly incubated eggs to provide additional genetic information such as parentage as well as retain the PVL for sperm observations (see also Morland et al., 2023).

We successfully genotyped 689 (97%) of the collected eggs with at least 10 microsatellite markers. Furthermore, we assigned parentage with high certainty to 98% of the eggs with known parents, thus showing unequivocally that we isolated offspring DNA. From this, we conclude that the amount of embryo DNA we obtained from the blastoderms is significantly higher than any potentially contaminating DNA. Our method of removing the blastoderm by scraping it off with a flattened pipette tip appears to be an effective technique to retrieve the embryo cells while minimising other cells such as maternal granulosa cells or sperm cells that may be present on the PVL.

The reliability of this method is, however, limited by the quality of the DNA that can be procured, which is influenced by the timing of material collection. When working with degraded or low amounts of DNA, genotyping errors may occur (Taberlet et al., 1996; Taberlet & Luikart, 1999). Such errors occurred seldom in our experimental eggs: only 7% of the eggs had additional alleles, 4% had a mismatch with the mother and two samples showed low amplification (genotyped at <10 loci). We account this to the fact that these eggs were fresh and viable and the blastoderms were easy to collect, resulting in high‐quality DNA. We found no difference in the quality of the DNA from experimental eggs that were incubated for 24 h compared to eggs that had not been incubated (broken eggs: *N* = 10, abandoned eggs: *N* = 136, and control eggs: *N* = 10).

Abandoned eggs also showed relatively low rates of genotyping errors. Eggs abandoned before the onset of incubation have little embryonic development but can remain stable in this state for many weeks (Birkhead et al., 2008; Burley & Vadehra, 1989). Only a small proportion of the abandoned eggs (6%, *N* = 13) could not be genotyped at 10 or more loci, with three eggs showing no amplification at all despite having sperm and cells on the PVL. The lack of amplification may have been due to difficulties with collecting the blastoderm, degraded embryonic material or problems during the DNA extraction.

Unhatched eggs showed the highest rate of mismatches as well as additional alleles, presumably due to lower quality DNA after 14 days of incubation plus 5 days after the rest of the clutch had hatched. Elevated temperatures during incubation may have led to degradation of the limited number of embryo cells. The blastoderms we processed from unhatched eggs most likely died shortly after laying.

Across all egg types, we sporadically detected additional alleles (3–4 instead of 2). In most cases, the two alleles from one or both parents were present, indicating a low level of contamination from sperm or from maternal cells (see Arnold, Orr, & Griffiths, 2003; Aslam et al., 2012; Morland et al., 2023). Additional alleles that could not be accounted for may be artefacts generated by the PCR due to the increase in the number of cycles required to detect small amounts of DNA or may stem from sperm from extra‐pair males.

Mismatches with the presumed genetic parents were usually one repeat unit longer or shorter than the expected allele, suggesting that they were caused by slippage during the PCR (Taberlet et al., 1996), or—in rare cases—by a mutation event (Ellegren, 2004).

Approximately one‐tenth of eggs in wild birds fail to hatch and embryo mortality occurs most frequently during early development, that is, before it can be detected by macroscopic examination (Hemmings & Evans, 2020). Our method would be most valuable for such eggs to give insight into the causes of hatching failure and early embryo mortality. Although our sample size of unhatched eggs was small (*N* = 10), we were still able to genotype eight of these eggs for at least 10 loci and assign parentage. We therefore conclude that this method can be applied to unhatched eggs (see also Morland et al., 2023).

Some species, particularly endangered or threatened species often have high rates of unhatched eggs (Marshall et al., 2023; Savage et al., 2021). Obtaining information from unhatched eggs in such species is relevant for conservation and may help avoid low hatching success in captive breeding programs. Our method makes it possible to determine not only if an egg is fertilised, but also who the genetic parents are, which could give insight into genetic incompatibility issues or partner choice. Determining the underlying causes of hatching failure in such eggs may boost success in conservation management. For example, knowledge of the paternity of fertilised but undeveloped eggs in artificial insemination trials such as those carried out in the ka¯ka¯po¯ *Strigops habroptilus* breeding program (Savage et al., 2021) could help optimise mate (sperm) selection and fertilisation attempts (see also Croyle et al., 2015). Sperm numbers on the PVL are an indicator of the likelihood of the development of an embryo (Hemmings & Birkhead, 2015) and can therefore provide additional insight into causes of egg failure. While previous studies have focused on determining fertility status or sexing of unhatched, undeveloped eggs, our results demonstrate that considerably more information can be obtained from such eggs.

## AUTHOR CONTRIBUTIONS

**Kim Teltscher:** Conceptualization (lead); methodology (lead); writing – original draft (lead); writing – review and editing (supporting). **Bart Kempenaers:** Conceptualization (supporting); funding acquisition (lead); supervision (lead); writing – original draft (supporting); writing – review and editing (lead).

## FUNDING INFORMATION

This work was funded by the Max Planck Society.

## CONFLICT OF INTEREST STATEMENT

The authors declare that they have no competing interest.

## Supporting information


Data S1.

## Data Availability

All data used for this study are available in an Excel file in the Open Science Framework https://doi.org/10.17605/OSF.IO/DTZNV.
